# Public health aspects of snakebite care in West Africa: perspectives from Nigeria

**DOI:** 10.1186/1678-9199-19-27

**Published:** 2013-10-17

**Authors:** Abdulrazaq G Habib

**Affiliations:** 1 Infectious and Tropical Diseases Unit, Bayero University Kano, Kano, Nigeria

**Keywords:** Antivenom, Carpet viper, Envenoming, Hub-and-spoke, Nigeria, Snakebite

## Abstract

Snakebite envenoming is a major public health problem among rural communities of the Nigerian savanna. The saw-scaled or carpet viper (*Echis ocellatus*) and, to a lesser extent, the African cobras (*Naja* spp.) and puff adders (*Bitis arietans*) have proved to be the most important cause of mortality and morbidity. The main clinical features of *E. ocellatus* envenoming are systemic hemorrhage, incoagulable blood, shock, local swelling, bleeding and, occasionally, necrosis. Bites may be complicated by amputation, blindness, disability, disfigurement, mutilation, tissue destruction and psychological consequences. Antivenom remains the hallmark and mainstay of envenoming management while studies in Nigeria confirm its protection of over 80% against mortality from carpet-viper bites. However, the availability, distribution and utilization of antivenom remain challenging although two new antivenoms (monospecific EchiTab G and trispecific EchiTab ICP-Plus) derived from Nigerian snake venoms have proven very effective and safe in clinical trials. A hub-and-spoke strategy is suggested for broadening antivenom access to endemic rural areas together with instituting quality assurance, standardization and manpower training. With the advent of antivenomics, national health authorities must be aided in selecting and purchasing antivenoms appropriate to their national needs while manufacturers should be helped in practical ways to improve the safety, efficacy and potential coverage against snake venoms and pricing of their products.

## Introduction

Snakebite envenoming comprises a major public health problem among communities of the savanna region of West Africa, notably in Benin, Burkina-Faso, Cameroon, Ghana, Nigeria and Togo [1-4]. The precise incidence of snakebite is difficult to determine and is often grossly underestimated but a global reappraisal estimated the occurrence in the West African region of 10,001 to 100,000 snakebite envenomings with an incidence of 8.9-93.3/100,000 persons per year with an estimated 1001 to 10,000 deaths and a mortality rate of 0.5-5.9/100,000 persons per year [5]. A more recent study using a meta-analytic approach estimated that over 314,000 bites, 7,300 deaths and nearly 6,000 amputations occur from snakebites annually in Sub-Saharan Africa [6].

## Review

## Overview of snakebites in Nigeria

**Figure 1:**
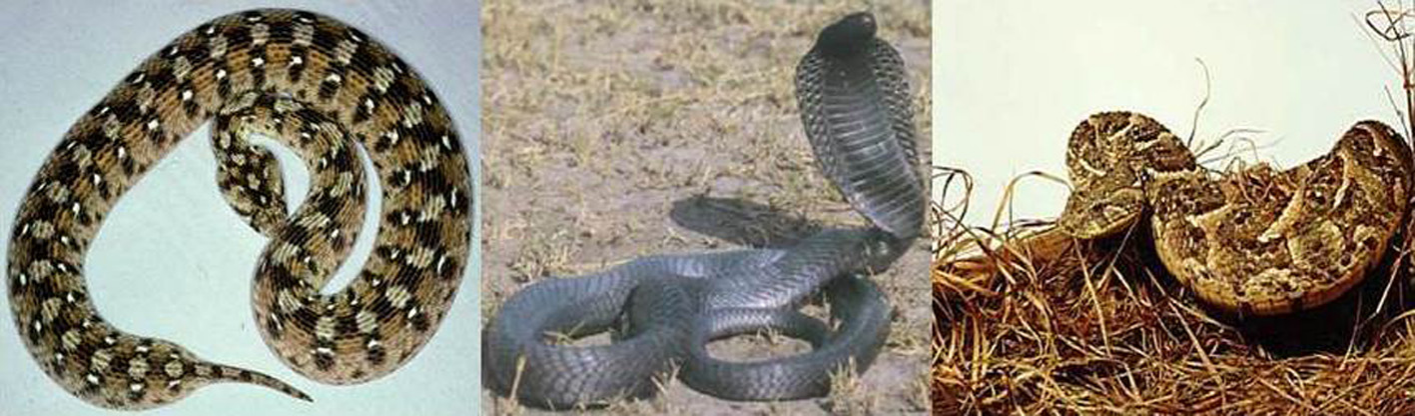
Carpet viper, African spitting cobra and puff adder.

In parts of the Nigerian savanna snakebite victims may occupy over 10% of hospital beds. In the Benue valley of Nigeria, the estimated incidence is as high as 497 per 100,000 population per year with 10 to 20% comprising untreated fatalities [1,7]. The saw-scaled or carpet viper (*Echis ocellatus*) has proved to be the most important cause of snakebite mortality and morbidity in the region (Figure 1).

The African cobras (*Naja* spp.), puff adders (*Bitis arietans*) (Figure 1) and mambas (*Dendroaspis* spp.) are frequently involved in attacks on humans while *Atractaspis* spp. and small vipers are only occasionally involved [1,8-11]. Snakebite affects farmers, nomads and rural dwellers of all ages. The main clinical features of *E. ocellatus* envenoming are systemic hemorrhage, incoagulable blood, shock, local swelling, bleeding and occasionally necrosis [1,8,12]. All body systems may be affected; cardiac and hemodynamic abnormalities may result while the strongest predictor of mortality is central-nervous-system involvement with intracranial hemorrhage [13-15]. Neurotoxicity has been reported following Egyptian cobra (*Naja haje*) bites in certain parts of the country. Occasionally, snakebite may lead to important complications such as amputation, blindness resulting from spitting cobra (*Naja nigricollis*) venom, opthalmia, fetal loss, and wound infection, tetanus and scarring with potential for malignant transformation, and psychological consequences e.g., excessive anxiety, stress, hysteria and worry [16-19].

## Economic impact of snakebite

The burden of human suffering caused by snakebites has been greatly underestimated, ignored and neglected for far too long. Snakebites, common in rural areas of many tropical developing countries including Nigeria, mainly affect the youth or agricultural workers who lack a political voice that effectively represent their needs. From the foregoing, it is evident that several thousand Nigerians fall victims of snakebites annually. These are mostly farmers, herdsmen and their rural-dwelling families. Each bite, whether or not accompanied by envenoming, leads to loss of work days. The high-risk groups are also the economically active population, so that their prolonged or short-term incapacitation at periods which coincide with the most intense farming activities can only lead to reduced agricultural production and low economic performance [20].

Furthermore, a recent study of 109 snakebite victims showed through multivariate analysis that delayed presentation beyond 24 hours after the bite (adjusted OR: 5.8; 95% CI: 2.0-17.0) and hospital stay over two days (adjusted OR: 19.5; 95% CI: 2.0-192.3) constitute independent risk factors for high-cost care [21]. Apart from loss of work days, the victims and their families have to bear the cost of treatment which can be quite high. It is not unusual for families to sell one or more cattle or a substantial portion of their harvest to pay for such treatment. In addition, a variable but unacceptably high percentage of victims die or are left with permanent mutilation each year. This constitutes a major liability to the families, since the victims are usually the breadwinners whose absence constitutes a permanent loss to the manpower resources available for the agricultural sector [20]. Even the fear of snakebite keeps many people away from such economic activities as peri-domestic poultry farming, a favorite haunt of *Naja nigricollis* or from working late on the farm for fear of being bitten on the way home in the darkening hours of the evening.

Recently, snakebite-induced mortality was shown to be inversely correlated with the Human Development Index, the per capita government expenditure on health and gross domestic product per capita, and directly associated with the percentage of the labor force in agriculture. It was further shown that snake envenoming is negatively associated with governmental expenditure on health, thus reaffirming its status as a disease of the poor [22]. Clearly therefore, poverty predisposes to snakebite and it is not only a major health problem but it is also a major impediment to economic prosperity.

Future health economic studies should factor in not only the burden of mortality but also the consequences resulting from amputation, blindness, disability, disfigurement, mutilation and tissue destruction [16-19]. In particular studies should derive quantitative estimates of economic and productivity losses by computing disability-adjusted life years (DALYs) and quality-adjusted life years (QALYs).

## First-aid and pre-hospital care

First aid measures include reassurance of patients and immobilization of the bitten limb with a splint or sling. First aid administrators should hasten the transfer of patients to a hospital along with the dead snake if found. Avoid harmful and time-wasting procedures such as incisions for application of native herbs, ice packs or electric shock, which have not yet been confirmed to be effective in controlled studies [23]. Avoid the use of tourniquets, constricting bands etc., as they have been shown to have minimal or no beneficial effect unless the snake was identified as dangerously neurotoxic, such as *N. haje* and *Dendroaspis* spp., since it may worsen ischemia and necrosis [24].

Recently, pre-hospital practices of 72 consecutive snakebite victims at a hospital in north central Nigeria were reported. The primary outcome assessed was death or disability at hospital discharge. Victims were predominantly male farmers, and in 54 cases (75%) the snake was identified as a carpet viper (*Echis ocellatus*), with the remainder unidentified. Most subjects (n = 58, 81%) attempted at least one first aid measure after the bite, including tourniquet application (n = 53, 74%), application (n = 15, 21%) or ingestion (n = 10, 14%) of traditional concoctions, bite site incision (n = 8, 11%), black stone application (n = 4, 5.6%), or suction (n = 3, 4.2%). The majority (n = 44, 61%) presented late (after four hours). Most (n = 53, 74%) were fully recovered at hospital discharge. Three deaths (4.2%) and thirteen (18%) disabilities (mainly tissue necrosis) occurred. The use of any first aid was associated with a longer hospital stay than no use (4.6 ± 2.0 days versus 3.6 ± 2.7 days, respectively, p = 0.02). The antivenom requirement was greater in subjects who had used a tourniquet (p = 0.03) and in those who presented late (p = 0.02). Topical application (OR: 15, 95% CI: 1.4-708) or ingestion of traditional concoctions (OR: 20, 95% CI: 1.4-963) was associated with increased risk of death or disability. Ingestion or application of concoctions was associated with a longer time interval before presentation, a higher cost of hospitalization, and an increased risk of wound infection [25].

All victims should be admitted to hospitals for at least 24 hours except in the case of a clearly non-venomous bite where the snake has been reliably identified. Pain may be managed with either oral paracetamol or narcotics. Persistent vomiting may be treated with intravenous chlorpromazine or other antiemetics. Intramuscular injections should be avoided because of susceptibility to hematoma formation in carpet viper bites.

## Antivenoms in Nigeria

Studies in Nigeria confirm that antivenom, which remains the mainstay in the management of envenomings, confers protection of over 80% against mortality from carpet viper bites [26]. However, in the last twenty years, a crisis in antivenom supply to sub-Saharan Africa has become evident [27,28]. The causes are complex but involve: a lack of commercial incentives for the companies that used to produce anti-venoms for Africa and inefficient distribution channels within the national health systems; ignorance of true antivenom requirements due to poor epidemiological information; the high cost of some products which make them unaffordable to local health systems; and loss of confidence in antivenoms, due partly to the marketing of ineffective and inappropriate products imported from overseas [27,29-31]. All these elements have contributed to a descending spiral of antivenom supply in sub-Saharan Africa and rising snakebite mortality and morbidity.

The gravity of this situation has prompted a growing concern. Since the 1990s, the Nigerian Ministry of Health has become increasingly aware of the medical importance of snakebite and the crisis in antivenom supply. Nigeria-UK collaborations were initiated to provide effective antivenoms. This resulted in the formation of the EchiTAb Study Group (Nigeria-UK) which, in 1995, developed the original EchiTAb (MicroPharm), an ovine Fab monospecific antivenom against Nigerian *E. ocellatus* venom. This antivenom was clinically tested and found to be effective, but because of its association with the problem of recurrent symptoms of envenoming, work continued to develop an antivenom with a more durable effect [12]. Currently, EchiTAb-G (a monospecific antivenom manufactured by Micropharm) and EchiTAb-Plus-ICP (a trispecific antivenom manufactured by Instituto Clodomiro Picado, University of Costa Rica) have been developed against venoms of Nigerian snakes [32].

In recently conducted clinical trials in Kaltungo hospital, Nigeria, comparing the two antivenoms in 400 *E. ocellatus*-envenomed patients with incoagulable blood, the antivenoms were found to be acceptably safe and durably effective [33]. At six hours, none of the participants had died and clotting had been permanently restored in 76% and 83% of patients on EchiTAb-G and EchiTAb-Plus-ICP, respectively. Further developments in response to the crisis include the commitment of several manufacturers to produce antivenoms for the region. Thus, in addition to laboratories that have previously manufactured antivenoms for Africa, such as EgyVac (Egypt), Sanofi-Pasteur (France) and South African Vaccine Producers, new manufacturers have entered the market including MicroPharm (UK) and Instituto Clodomiro Picado (Costa Rica), and the Instituto Bioclon (Mexico) [32,34]. This expansion greatly improves the prospects for better treatment of snakebite envenoming.

## Access to effective antivenom

Use of inappropriate or ineffective antivenoms following envenoming independently predicts mortality [15,30,31]. It is necessary that the regulatory authorities in the countries of the region ensure the procurement and distribution of appropriate antivenoms raised against locally prevalent snakes. Unscrupulous marketing of inappropriate products has had disastrous consequences. The use of such antivenoms has increased mortality and conversely, regionally appropriate antivenoms has reduced mortality by 70 to 90% [15,30].

Indeed, a delay in administering effective anivenom predicts the likelihood of dying; an hour’s delay before antivenom administration is associated with a 1% increased risk of dying [15]. The following three models of delay in maternal mortality may indeed be similar and applicable to snakebite: (1) delay in seeking suitable medical help for snakebite emergency for reasons of cost, lack of recognition of an emergency, poor education, lack of access to information and gender inequality; (2) delay in reaching an appropriate facility for reasons of distance, infrastructure and transport; (3) and delay in receiving adequate care when a facility has been reached due to shortages in qualified staff or unavailability of medical supplies (e.g., reliable antivenoms).

While distance from bite location to facilities with available stock of effective antivenom is not the sole determinant of delay, it is probable that distributing and providing antivenoms in relatively inaccessible rural endemic areas might reduce the distance travelled by victims and improve access to care [15].

**Figure 2:**
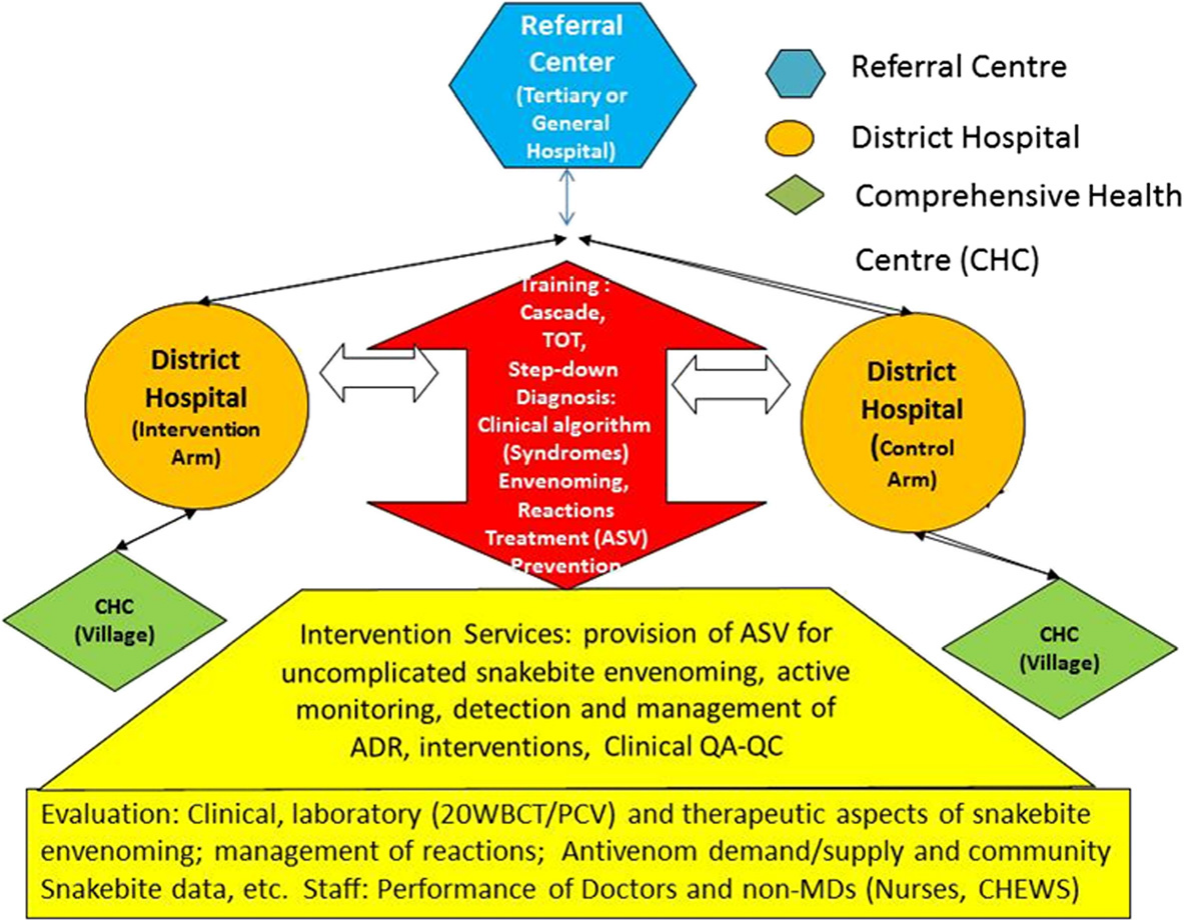
**Hub-and-spoke model for snakebite care.** Full meaning of acronyms: ADR: Adverse Drug Reactions; ASV: Anti-Snake Serum AntiVenom; CHC: Comprehensive Health Centre; CHEW: Community Health Extension Worker; MD: Medical Doctors; PCV: Packed Cell Volume; QA-QC: Quality Assurance Quality Control; TOT: Training of Trainers; 20WBCT: 20 minute Whole Blood Clotting Test.

A possible strategy for improving access would be to consider a hub-and-spoke model (also referred to as cluster model) for snakebite care and treatment. In this model, a secondary or higher-level hospital would be the hub while pairs of health facilities 20 to 30 km away (a district hospital and nearby village comprehensive health center) would constitute the spokes [Figure 2]. Attempts should be made to identify all patients that only seek traditional medicine practitioners and those that die in the community and link them with peripheral facilities. These lower level facilities (which normally do not stock antivenom) would thence provide and administer antivenoms based on case management protocol (CMP). This proposal includes technical oversight from the higher facility at the hub which would include step-down trainings, standardizations and the use of CMP by a lower cadre of staff often with no medical doctor in such rural facilities.

Given that antivenoms also have the potential to provoke anaphylactic reactions and serum sickness, it is important that CMP balances treatment information on use of antivenom, its benefit, and the detection and management of adverse reactions. Antivenoms and complementary items (analgesics, tetanus toxoid and antisera, parenteral fluid, wound care consumables etc.) should be made available regularly in health care centers rather than by demand. All envenomed patients presenting at these facilities, together with their clinical features, management and outcome should be recorded prospectively over time, thereby improving the epidemiological data gathered on the rural snakebite burden (Figure 2).

Subsequently, the hub-and-spoke strategy should be evaluated in a community cluster randomized controlled trial in which some pairs are randomly selected to receive a training program to manage uncomplicated snakebite, administer antivenom, as well as detect and manage adverse reactions. This will serve as the intervention arm while other pairs will serve as a comparator and offer the current standard of care, i.e. refer snake-bitten patients to secondary or higher-level facilities for care. Ultimately, such a study will allow determination of the effectiveness of antivenoms in routine practice in villages (as against efficacy estimated from clinical trials); the effect of reducing delay on outcome; adverse drug reactions detection in villages by lower-level health care workers; the cost savings to victims; and logistical issues in antivenom distribution/replenishment at lower levels of the health system as a prelude to adoption of the strategy for maintaining a nationwide supply of antivenom (Figure 2).

## Quality assurance, standardization and training

Even in the presence of appropriate and effective antivenom, the importance of education, standardization, training and overall quality assurance should not be underestimated. In a study conducted in Mathias Hospital, Yeji, northern Ghana, snakebite cases were an important cause of morbidity and mortality with a case fatality rate of 11% (8/72). But prior to instituting intervention measures, case management difficulties included uncertainty about the assessment of the envenoming severity, the antivenom dosage and the response to treatment. Subsequently, an intervention package comprised of several components including development of a treatment protocol, staff training, monitoring of compliance and patient education was introduced.

During a 33-month post-intervention period there was excellent protocol compliance, fewer snakebite complications, and a fall in the mortality rate to 1.3% (3/238), that is, a 90% reduction compared with a 15-month baseline review. There was a 50% increase in snakebite admissions and fewer delays. The authors concluded by recommending a similar quality assurance strategy, involving case review and the use of a treatment protocol with monitoring of compliance to sustain improved snakebite outcomes in comparable settings, particularly if inexperienced staff are involved in care [4]. Such standardization approaches should be widely introduced in areas endemic for bites.

## Conclusions

## Prevention and future perspectives

As a preventive measure, protective clothing, including boots and long trousers, should be worn whilst working in snake-infested areas. Improved techniques of effective and safer antivenom production (e.g., whole immunoglobulin versus digested fragments) and best formulation with extended shelf life (freeze-dried versus liquid formulation) in an African setting should be critically explored.

The field of proteomics (venomics, antivenomics) will continue to complement preclinical studies and clinical trials in evaluating antivenoms for use in the region. The proteomics approach brings with it the potential to design new immunizing mixtures from which to raise potent antivenoms with wider therapeutic coverage/ranges [35]. Already, activity spectra of coverage of certain antivenoms (EchiTab ICP-Plus) raised against Nigerian snakes have been confirmed as extending to other species by means of antivenomics [36]. As only about 8.5% of snakebite victims attend hospitals in Nigeria people should be educated and enlightened about the benefits of orthodox medicine so as to reduce the resulting morbidity and mortality [37].

Certain herbs in Nigeria (*Aristolochia albida, Guiera senegalensis, Schumaniophyton magnificum* etc.) were found to act on snake venoms in experimental animals but studies are ongoing and remain inconclusive [38-40]. Their use as accessory and supplementary agents should be evaluated further.

## Recommendations

Expert advice and encouragement must be offered to Nigerian health authorities, regulatory agencies, antivenom producers and medical personnel to improve every aspect of snakebite treatment. In particular:

● Antivenom manufacturers must be helped in practical ways to improve the safety and efficacy of their products. This requires the design and implementation of long-term technology transfer programs involving both North–south (i.e., collaborations between institutions in developed and developing countries such as between the United Kingdom and Nigeria) and South-South partnerships (i.e., collaborations between two institutions in the developing world such as between institutions in Nigeria and Brazil or Costa Rica).

● National health authorities must be helped to select and purchase antivenoms appropriate to their national needs.

● Adequate policies for antivenom distribution must be developed in the country and in each health zone (or state) in order to provide antivenoms where they are most needed. There should be sufficient political will to prevent any exhaustion of antivenom stock, even a temporary one.

● Medical personnel must be trained in the modern management of snakebites and, most crucially, in the selective use of antivenoms through the use of educational pamphlets, as well as lectures and practical demonstrations.

● Undergraduate and post-graduate training of medical students, nurses and community health care workers should incorporate ophidian, scorpion and spider envenoming in their curriculum.

● Communities must be informed and educated about snakebite risks through the use of posters and leaflets. They should be offered realistic solutions that mitigate the hazards and empower the people themselves to help manage the problem in practical and sustainable ways.

● Research directed at improving the available methods of first aid, primary clinical care and patient rehabilitation must be accorded priority and funded at national and state levels.

● Surveillance and reporting systems that enable collation of reliable epidemiological and clinical data need to be developed, tested and implemented; and the data used to support rational resource allocation and distribution, and appropriate prioritization of snakebite envenoming as a neglected tropical disease at all levels.

## Competing interests

The author declares that there are no competing interests.
